# Generation of ^34^S-substituted protein-bound [4Fe-4S] clusters using ^34^S-L-cysteine

**DOI:** 10.1093/biomethods/bpy015

**Published:** 2019-01-22

**Authors:** Jason C Crack, Melissa Y Y Stewart, Nick E Le Brun

**Affiliations:** Centre for Molecular and Structural Biochemistry, School of Chemistry, University of East Anglia, Norwich Research Park, Norwich, NR47 TJ, UK

**Keywords:** iron–sulphur, cysteine, mass spectrometry, resonance Raman spectroscopy

## Abstract

The ability to specifically label the sulphide ions of protein-bound iron–sulphur (FeS) clusters with ^34^S isotope greatly facilitates structure–function studies. In particular, it provides insight when using either spectroscopic techniques that probe cluster-associated vibrations, or non-denaturing mass spectrometry, where the ∼+2 Da average increase per sulphide enables unambiguous assignment of the FeS cluster and, where relevant, its conversion/degradation products. Here, we employ a thermostable homologue of the *O*-acetyl-l-serine sulfhydrylase CysK to generate ^34^S-substituted l-cysteine and subsequently use it as a substrate for the l-cysteine desulfurase NifS to gradually supply ^34^S^2−^ for *in vitro* FeS cluster assembly in an otherwise standard cluster reconstitution protocol.

## Introduction

Proteins that contain iron–sulphur (FeS) clusters are extremely widespread in nature and play key roles in an array of biochemical processes from respiration and photosynthesis to DNA replication. They contain iron and inorganic sulphide in structural arrangements that differ in nuclearity as well as shape, for example, the rhombic [2Fe-2S] and cubic [4Fe-4S] clusters [1]. Cysteine thiolates (RS^−^) are by far the most common amino acid ligands to FeS clusters, but other residues such as histidine (–N=), serine (R–O^−^) and aspartate (R–CO_2_^−^) are known [2]. In addition to roles in electron transfer, redox and Lewis acid catalysis, the inherent reactivity of FeS clusters with a range of small molecules makes them ideal candidates for sensing environmental changes and stresses caused by reactive oxygen (ROS) and/or nitrogen (RNS) species. FeS cluster containing transcriptional regulators have evolved to exploit this sensitivity as a way of modulating protein–DNA interactions and hence a means to effect transcriptional regulation [3]. Recent advances in the purification and handling of extremely sensitive FeS proteins at high concentration have facilitated the application of a range of biophysical techniques to study the nature and reactions of the FeS cluster with ROS/RNS. The use of stable isotopes, particularly of iron (^57^Fe), sulphur (^34^S), and nitrogen (^15^N in NO) have proved instrumental in these advancements, for example, via mass spectrometry and nuclear resonance vibrational spectroscopy (NRVS) [4–8].

We previously described a method for the *in vivo* incorporation of ^57^Fe into FeS clusters [9]. However, it is not cost-effective to attempt large-scale ^34^S-labeling *in vivo*. Fortuitously, in many cases, FeS clusters will self-assemble, *in vitro*. Addition of ferrous and sulphide salts is one approach, which works well in some cases but can be difficult to control, leading to black FeS precipitates or adventitious, non-physiological protein-associated FeS species. A more refined biochemical method that is widely used by researchers in the FeS protein field employs an enzyme naturally involved in FeS cluster assembly *in vivo* [10]. The simplest of these utilizes a cysteine desulfurase, typically *Azotobacter vinelandii* NifS, as a way of gradually generating sulphide from L-cysteine [11–13]. NifS reconstitutions typically result in a good recovery of holo-protein (≥70%) with spectroscopic features indistinguishable from those of the *in vivo*-derived counterpart [10, 14].

Currently, ^34^S-L-cysteine is not commercially available. Two *in vitro* methods for the total synthesis of L-cysteine have been reported. The first, in which elemental sulphur is reacted with benzyl-magnesium chloride and L-β-chloroalanine prior to the formation of cysteine, provides poor yields of a crucial intermediate in the process, S-benzylcysteine [15, 16]. The second, involving the reaction of thioacetic acid with α-acetamidoacryl acid, results in a racemic mixture of d- and L-cysteine [17]. *In vivo*, L-cysteine is synthesized from L-serine by the action of the two enzymes CysE and CysK [18]. CysE (EC 2.3.1.30) is a L-serine *O*-acetyltransferase, while CysK (EC 2.5.1.47) is a pyridoxal-5′-phosphate dependent enzyme with *O*-acetyl-L-serine (OAS) sulfhydrylase activity, catalysing the stereo specific formation of L-cysteine via a nucleophilic addition of inorganic sulphide to OAS [19]. We note that naturally occurring D-cysteine is synthesized by a dedicated L- to d-amino acid racemase (EC 5.1.1.10) distinct from CysK.

Here, we build on the method of Ono *et al.* [20] for synthesizing ^34^S-L-cysteine from commercially available OAS by employing a thermostable CysK, from *Geobacillus stearothermophilus* [21], and demonstrate its utilization in the preparation of ^34^S-labeled FeS clusters in a number of FeS clusters containing transcriptional regulators.

## Materials and methods

### Preparation of CysK

Luria-Bertani medium (2 × 500 ml) was inoculated with freshly transformed BL21 λDE3 *Escherichia coli* containing the CysK expression vector [pET11a encoding a codon optimized *cysK* gene (uniprot id: Q84IF9) from the thermophile *G. stearothermophilus* cloned using *Nde*I and *Bam*HI sites; Genscript]. *Geobacillus stearothermophilus* CysK was selected because it has been previously characterized [21], and its thermal stability and general robustness provides enhanced flexibility for possible future applications. Ampicillin (100 µg/ml) was added and the culture grown at 37°C, 200 rpm, until OD_600 nm_ reached ∼0.8. Expression was induced by the addition of isopropyl β-D-1-thiogalactopyranoside (IPTG, 1 mM). After 20 min, the cultures were supplemented with 50 µM L-methionine, 50 µM glycine, 50 µM (NH_4_)_2_SO_4_ and Minimum Essential Medium (MEM) vitamins (10 µl/ml, containing pyridoxal hydrochloride; a precursor to pyridoxal-5′-phosphate, 100×, Sigma Aldrich) and incubated for a further 4 h at 37°C. The cells were harvested by centrifugation at 10 000×*g* for 15 min at 4°C. Cell pellets were resuspended in 40 ml of buffer A (50 mM Tris HCl, pH 7.0) and sonicated twice while on ice, and then centrifuged at 40 000×*g* for 45 min at 1°C. The cleared cell lysate was treated with streptomycin sulphate (15 mg/ml), incubated on ice for 30 min, and then centrifuged at 20 000×*g* for 15 min at 1°C. The supernatant was then fractionally precipitated with ammonium sulphate between 50% and 80% saturation [22], before centrifugation at 20 000×*g*, as described above. The precipitate from the 80% saturation step was dissolved in 15 ml of buffer A and dialysed (10 kDa MWCO) overnight at 4°C against 1 L buffer A.

Post dialysis, the sample was loaded onto a Q Sepharose column (15 ml) and washed with buffer A containing 3% (v/v) buffer B (50 mM Tris HCl 800 mM NaCl, pH 7.0). Bound proteins were eluted using a 50 ml linear gradient between 3% and 32% (v/v) buffer B. Fractions (1 ml) containing CysK were pooled, incubated at 65°C for 30 min, centrifuged at 17 000×*g* for 15 min at room temperature, diluted 5-fold with buffer A and concentrated, as previously described [9], using a Q Sepharose column (1 ml). CysK was eluted using buffer B, separated into 50 µl aliquots and stored at −80°C until needed. Purity of the final preparation was ∼95%, as judged by SDS–PAGE. Protein concentration was determined by the method of Bradford (BioRad) [23], with bovine serum albumin as the standard.

### Preparation of NifS

*Azotobacter vinelandii* NifS was purified largely as previously described [11]. Briefly, *E. coli* BL21(DE3) cells containing the *nifS* expression plasmid pDB551 were grown at 37°C, 200 rpm in Luria broth containing ampicillin (100 mg/l). NifS production was induced when cells reached A_600 nm_ = 0.6 by the addition of 1 mM IPTG. Cultures were supplemented with 1× MEM vitamins 10 min post induction and further incubated for 2 h. Following harvesting, cell pellets were resuspended in Tris buffer (25 mM Tris pH 7.4), lysed by sonication and centrifuged. Solid streptomycin sulphate was added to the supernatant (1.5 g/100 ml), which was incubated on ice for 15 min. The resulting suspension was centrifuged and the supernatant fractionated using ammonium sulphate, with NifS precipitating in the 25–45% cut. NifS was dissolved in Tris buffer, dialysed (10 kDa MWCO) overnight at 4°C against 1 l of Tris buffer. Post diaslysis, the sample was loaded on to a 10 ml HiTrapQ Sepharose column (GE Healthcare) and eluted using a 0.1–0.6 M NaCl gradient, yielding NifS at ∼95% purity, as judged by SDS–PAGE. The sample was aliquoted (20 µl) and stored at −80°C until needed. Protein concentration was determined as above.

### Reduction of S^0^ to sulphide

^34^S-sulphur (98% enrichment, Cambridge Isotope Laboratories, Goss Scientific) was reduced to sulphide (S^2^^−^) by the action of sodium metal in liquid ammonia via a Schlenk line as previously described [24, 25]. Briefly, an aliquot (∼135 mg) of sodium metal was dissolved in liquid ammonia. To this was added an aliquot (∼100 mg) of ^34^S-sulphur, maintaining a molar stoichiometry of approximately two sodium per sulphur. During the reaction the blue colour typical of solvated electrons faded, after which the liquid ammonia was allowed to evaporate under a stream of nitrogen. The remaining residue was carefully dissolved in a minimal volume of anaerobic 25 mM NaOH and passed through a 0.2 µm filter to remove any particulates. The resulting sulphide solution was assayed according to the method of Beinert [26].

### Synthesis of ^34^S-L-cysteine

To generate ^34^S-L-cysteine, 1 ml aliquots of OAS and TCEP dissolved in buffer C (200 mM HEPES pH 7.5) were combined with ^34^S-sulphide and buffer C in 4 ml head space-less vial together with 200 µl of 12.5% (w/v) NaOH. The final reaction mixture contained 134 mM OAS, 67 mM sodium sulphide, 25 mM TCEP, 75 mM NaOH and had a pH of ∼7.5, as judged by indicator paper. An aliquot (50 µl, 0.4 mg/ml final protein concentration) of CysK was added and the reaction mixture incubated at 50°C overnight. After cooling, the reaction mixture was diluted to 20 ml with 100 mM HEPES pH 7.5, passed through a 0.2 µm filter and then a 1 ml Q Sepharose column to remove CysK. The eluent was devoid of protein [23] and sulphide [26] but reacted readily with 5,5′-dithiobis(2-nitrobenzoic acid) (DTNB) [27], confirming complete conversion of sulphide to cysteine. This solution was used without further purification in FeS cluster reconstitution reactions (see below). Where necessary, reaction mixtures were analysed by thin layer silica gel chromatography, with a propan-2-ol, acetic acid, water (8:1:1) solvent system, as previously described [28]. The plates were visualized with ninhydrin [0.2% (w/v) in acetone/ethanol (9:1)] or DTNB [0.1% (w/v) in water/ethanol (9:1)]. L-cysteine, L-cystine and OAS served as TLC standards, giving R_f_ values of 0.29, 0.13 and 0.54, respectively [27–29].

### FeS cluster reconstitution

FeS proteins (*E. coli* FNR, *Streptomyces coelicolor* NsrR and *Rhizobium leguminosarum* RirA) were purified as previously described [4–7]. Naturally incorporated FeS clusters were removed by dialysis in the presence of air. Reconstitution of the FeS cluster was carried out with NifS, as previously described [9], except that ^34^S-L-cysteine solution was used in place of natural abundance L-cysteine. Briefly, anaerobic apo-protein (in the range 70–500 µM) was treated with a 16-fold excess of ^34^S-L-cysteine, 40-fold excess of dithiothreitol, and up to a 10-fold excess of (NH_4_)_2_Fe(SO_4_)_2_ over the apo-protein. The buffer used was dependent on the protein: FNR, 25 mM HEPES, 2.5 mM CaCl_2_, 100 mM NaCl, 100 mM NaNO_3_, pH 7.5; NsrR, 50 mM Tris, 50 mM NaCl, 5% (v/v) glycerol, pH 8.0; RirA, 25 mM HEPES, 2.5 mM CaCl_2_, 50 mM NaCl, 750 mM KCl, pH 7.5. *Azotobacter vinelandii* NifS (∼225 nM) was added and the reaction mixture incubated with stirring at 20–37°C, depending on the protein (FNR, 37°C; NsrR, 30°C; RirA, 20°C). UV–visible absorbance spectra were recorded every 20 min until no further increases in absorbance due to the FeS cluster were apparent (the time required varies between proteins but is typically complete after a few hours). Low molecular mass contaminants were removed by applying the reconstitution reaction mixture to a 1 ml HiTrap heparin column (GE Healthcare) and eluting with a NaCl gradient of 100–500 mM in the same buffer as above. For non-DNA-binding FeS proteins, a Sephadex G25 column (PD10, GE Healthcare) can be used instead.

### Spectroscopy and mass spectrometry

UV–visible absorbance measurements were made using a Jasco V500 spectrometer and circular dichroism (CD) spectra were measured with a Jasco J810 spectropolarimeter. The [4Fe-4S]^2+^ cluster concentration was determined by absorbance spectroscopy using previously published extinction coefficients (mM^-1^ cm^-1^): FNR (406 nm), 16.22; NsrR (406 nm), 13.30; RirA (383 nm), 13.46 [4, 14, 30].

HPLC-MS experiments with cysteine were performed using an UltiMate 3000 HPLC system (Dionex, Sunnvale, CA, USA), and a Bruker micrOTOF-QIII electrospray ionization time-of-flight (TOF) mass spectrometer (Bruker Daltonics, Coventry, UK), in positive ion mode. LC-MS samples were brought to 80% (v/v) acetonitrile, loaded onto a Luna NH_2_ column (2 × 100 mm) (Phenomenex) and eluted (0.6 ml/ml) using a HILIC gradient between solvent A [95% (v/v) aqueous 5 mM ammonium formate pH 3.75, 5% (v/v) acetonitrile] and solvent B [95% (v/v) acetonitrile, 5% (v/v) aqueous 100 mM ammonium formate pH 3.75], as previously described [31]. Mass spectra were recorded using Bruker oTOF Control software with parameters as follows: dry gas flow 8.5 l/min, dry gas 200°C, nebulizer pressure 1.2 bar, capillary voltage 4500 V, offset 500 V, collision RF 400 Vpp. The spectrometer was calibrated with sodium formate in the 50–600 m/z range.

Electrospray ionization mass spectrometry (ESI-MS) of proteins under non-denaturing conditions in ammonium acetate buffer was performed using a Bruker microOTOF-QIII mass spectrometer operating in the positive ion mode and calibrated using ESI-L Low concentration tuning mix, as previously described [4–6]. Processing and data analysis were carried out using Compass Data Analysis version 4.1. Neutral mass spectra were generated using ESI compass Maximum Entropy deconvolution algorithm version 1.3. Exact masses are reported from peak centroids representing the isotope average neutral mass. For apo-proteins, these are derived from the m/z spectra, for which peaks correspond to [M + zH]/z. For cluster-containing proteins, where the cluster contributes charge, the peaks correspond to [M + (FeS)^n+^ + (z−n)H]/z, where M is the molecular mass of the protein, FeS is the mass of the FeS cluster of n+ charge, H is the mass of the proton and z is the total charge. In the expression, the n+ charge of the FeS cluster offsets the number of proteins required to achieve an observable charge state with z charges [32, 33]. Predicted masses are given as the isotope average of the neutral protein in which FeS cluster binding is expected to be charge compensated.

## Results and discussion

### Synthesis of ^34^S-L-cysteine by CysK

The ability to specifically isotopically label the acid labile sulphides of FeS clusters is a powerful tool for enhancing studies using spectroscopic techniques, such as resonance Raman spectroscopy and nuclear vibrational resonance spectroscopy. These methods probe vibrations involving sulphur species where the increase in mass due to incorporation of ^34^S results in a decrease in the energy of vibrational bands, enabling the deconvolution of cluster Fe-S and Fe-cysteinyl contributions to the vibrational spectrum [6, 7]. It is also extremely useful for studies by non-denaturing mass spectrometry, in which assignments of FeS clusters and their conversion/degradation products can be made unambiguously through the detection of mass shifts [5].

**Figure 1:**
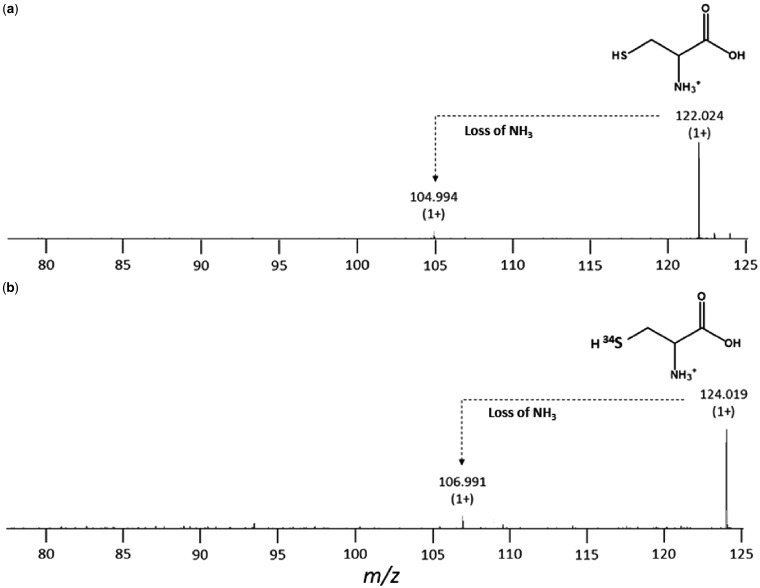
ESI-MS of natural abundance and ^34^S-L-cysteine. m/z spectra of (a) natural abundance L-cysteine with the major peak corresponding to the [M + H]^1+^ ion, along with a low intensity fragment, and (b) ^34^S-substituted cysteine containing the same peaks but shifted by approximately 2 g/mol. Cysteine was loaded onto the Luna NH_2_ column at 20 mM, while ^34^S-L-cysteine was at 0.4 mM. The ESI-MS data correspond to the cysteine peak eluted at 5.2 min.

The first step towards a simple method to achieve specific isotopic substitution of cluster sulphide was the synthesis of ^34^S-L-cysteine. This was achieved according to Ono *et al.* [20], except CysK from *G. stearothermophilus* was used to catalyse the reaction between ${\text{Na}}_{2}^{34}$S and *O*-acetyl-L-serine at 50°C overnight [6, 7]. After removal of enzyme, the reaction mixture contained no detectable protein or sulphide, indicating complete reaction and this was confirmed by reaction with DTNB, which produced the characteristic yellow colour indicative of the presence of thiolate species. TLC plates developed with DTNB yielded a single spot with a R_f_ value of 0.25 (± 0.03), which is consistent with that of L-cysteine (0.29). Plates developed with an amine-specific reagent, ninhydrin, revealed two spots with R_f_ values of 0.25 (± 0.03) and 0.51 (± 0.03), corresponding to cysteine and unreacted OAS, respectively. LC-MS of purified ^34^S-L-cysteine revealed a single major [M + H]^1+^ peak at m/z = 124.019, corresponding to a mass of 123.011 g/mol, very close to the expected monoisotopic mass of ^34^S-cysteine 123.016 g/mol ([M + H]^1+^ m/z = 124.023) and shifted by approximately +2 g/mol relative to that of natural abundance cysteine (Fig. 1). Addition of the reaction mixture to a sample of NifS caused the major absorbance peak at 392 nm to shift to 416 nm with the concomitant appearance of a band at 370 nm (not shown), consistent with the presence of cysteine, as previously described [11]. *O*-acetylserine alone did not alter the spectrum of NifS and we note that d-cysteine is not a substrate for the enzyme [11].

### Reconstitution of FeS clusters containing ^34^S-sulphide

The reconstitution of FeS clusters using the cysteine desulfurase NifS is well known to be more efficient than equivalent reactions employing a sulphide salt as the source of sulphur, presumably due to the gradual production of sulphide that minimizes formation of unproductive iron–sulphide precipitates [11–13]. Here, using a standard procedure with ^34^S-l cysteine in place of natural abundance cysteine, apo-protein forms of *E. coli* FNR [7], *S. coelicolor* NsrR [30], and *R. leguminosarum* RirA [4] were reconstituted to generate FeS cluster (holo) proteins. Resulting samples were typically ≥70% cluster loaded [5–7].

**Figure 2:**
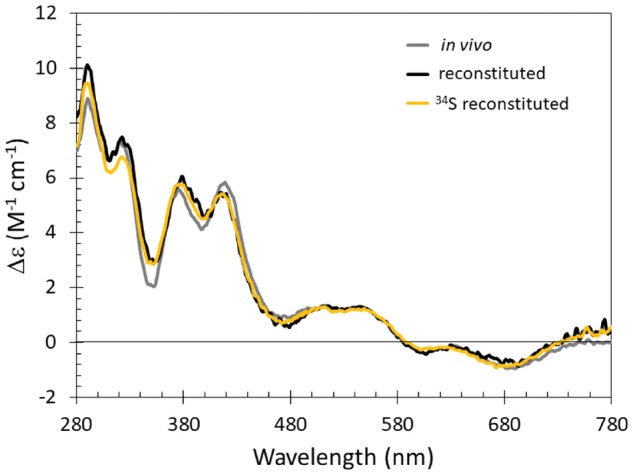
Comparison of native and reconstituted [4Fe-4S] FNR. Comparison of the CD characteristics of [4Fe-4S] S24F FNR assembled *in vivo* (grey line), or reconstituted *in vitro*, with (yellow line) and without ^34^S-L-cysteine (black line). The buffer was 25 mM HEPES, 2.5 mM CaCl_2_, 100 mM NaCl, 100 mM NaNO_3_, pH 7.5. Note that the S24F variant of FNR was employed in time resolved ESI-MS studies of the FNR cluster conversion mechanism [5].

It is important to note that FeS proteins readily form sulphur adducts, usually involving the insertion of cluster-derived sulphide that has undergone oxidation to form sulphane (S^0^), which in turn can incorporate into the thiolate side chain of cysteine residues to form persulphides adducts [5, 7, 34]. The preparation of *clean, persulphide-free* apo-protein is an important prerequisite for preparation of ^34^S-labelled FeS proteins (particularly when mass spectrometry studies are planned) and so Tris (2-carboxyethyl) phosphine (TCEP) was used here prior to reconstitution [35]. Post reconstitution, proteins were separated from low molecular weight species via a combination of weak ion exchange and/or gel filtration techniques; in some cases, this can selectively enrich the holo-protein content of the sample [9]. It is important to compare the biophysical properties of the *in vitro* reconstituted protein to those of *in vivo* assembled material, wherever possible [10, 14]. CD spectroscopy is ideally suited for this purpose, as the electronic transitions that underlie the broad absorption spectrum of many FeS proteins can be resolved via CD spectroscopy. This optical activity arises from the asymmetric protein fold to which the FeS cluster is ligated. The CD spectrum can be used to ensure the quality of the FeS protein samples between preparations, that is, for natural abundance and ^34^S-labelled preparations of the same protein. Figure 2 shows the anaerobic CD spectra of [4Fe-4S] FNR assembled *in vivo* and reconstituted *in vitro* with and without ^34^S-L-cysteine in place of regular cysteine. The CD spectra all display three major positive features at 330, 380 and 420 nm with comparable Δɛ values indicating the [4Fe-4S]^2+^ clusters in each preparation are in essentially identical environments [9].

### Mass spectrometric determination of ^34^S incorporation into FeS clusters

**Figure 3:**
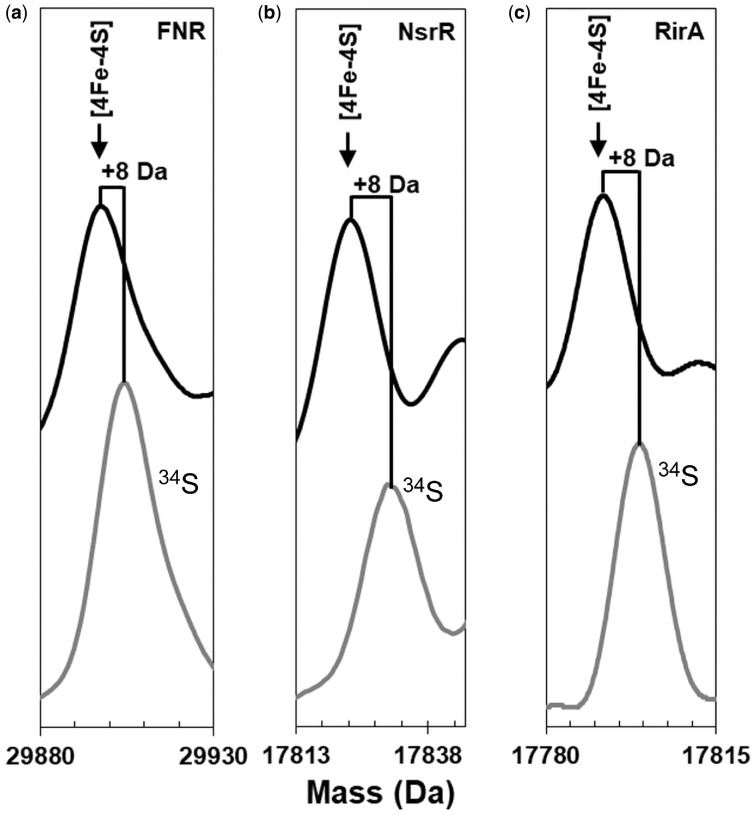
ESI-MS of ^34^S reconstituted FeS cluster proteins. Isotopically enriched samples of [4Fe-4S] (a) *E. coli* S24F FNR, (b) *S. coelicolor* NsrR, and (c) *R. leguminosarum* RirA were analysed by non-denaturing ESI-MS. The sulphides of naturally abundant [4Fe-4S] clusters (black line) were substituted by ^34^S sulphide (grey line), in each case giving an isotopic shift of +8 Da. Data for S24F FNR were previously published [5].

During ESI-MS biological analytes are introduced into the mass spectrometer in a non-denaturing volatile aqueous solvent, giving rise to multiply charged ions in the gas phase that preserve the non-covalent interactions found in protein-protein and protein-cofactor complexes [36–38]. This non-denaturing ESI-MS technique is finding increasing application in the characterization of a wide range of metalloproteins [39, 40], including a growing number of FeS proteins [4, 5, 32, 41]. The strength of the technique lies with its ability to identify, as well as determine the stoichiometry of, protein-associated metal (and sulphide) ions. We have recently applied time-resolved ESI-MS to the study of FNR, a master regulator controlling the switch between anaerobic and aerobic respiration in *E. coli* and many other bacteria [5]. In FNR, the [4Fe-4S] cluster functions as a sensory module, undergoing reaction with O_2_, leading to conversion to a [2Fe-2S] cluster with concomitant loss of high-affinity DNA binding. In this case, ESI-MS permitted the detection of cluster conversion intermediates and products, including [3Fe-4S], [3Fe-3S], [2Fe-2S], and the persulphide coordinated [2Fe-2S] clusters identified via resonance Raman [5, 7]. We note that ^32^S and ^16^O (in multiples of two) would have the same mass as a persulphide coordinated [2Fe-2S] cluster. Therefore, we utilized a ^34^S-labelled form of [4Fe-4S] FNR, see Fig. 3a. The ESI-MS of this sample contained a major peak at +8 Da compared to that of the natural abundance [4Fe-4S] FNR sample (29 905 vs 29 897 Da). The expected mass difference for replacement of all cluster sulphides (95% ^32^S) with ^34^S is +7.6 Da (taking into account the natural abundance of sulphur isotopes). To demonstrate further the general utility of the methodology, ^34^S substituted forms of two other FeS containing transcriptional regulators, *S. coelicolor* NsrR and *R. leguminosarum* RirA, were generated (Fig. 3b and c, respectively). In each case, full incorporation of ^34^S was demonstrated through the observation of a +8 Da mass shift compared to the mass observed for the protein containing a cluster with natural abundance sulphur. For NsrR, the [4Fe-4S] peak shifted from 17 823 to 17 831 Da (Fig. 3b); for RirA, the shift was from 17 792 to 17 800 Da (Fig. 3c). Note; for clarity, the mass range shown in Fig. 3 has been restricted to the area immediately either side of the main [4Fe-4S] protein peak in the monomeric region of the spectrum to highlight the ^34^S induced mass shift. Full mass spectra for FNR, NsrR, and RirA containing naturally abundant [4Fe-4S] clusters have been published elsewhere [4, 5, 30].

## Conclusions

Here, we describe a convenient and generally applicable method for specifically labelling the sulphides of FeS cluster proteins with ^34^S. The method, which is based on two enzyme-catalysed reactions, avoids the problems associated with direct chemical reconstitution of FeS cluster proteins by providing regulated amounts of ^34^S^2^^−^ for cluster assembly. The resulting ^34^S-labeled FeS clusters greatly facilitate structural and mechanistic studies, as already demonstrated through resonance Raman [7], NRVS [6], and ESI-MS studies [5].

## Author contributions

J.C.C. and N.E.L.B. designed experiments; J.C.C. and M.Y.Y.S. carried out the experimental work; J.C.C., M.Y.Y.S. and N.E.L.B. analysed the data; J.C.C. and N.E.L.B. wrote the paper.

## Data availability

Data available at DOI 10.17605/OSF.IO/XQRSN (6 December 2018, date last accessed).
